# Social Support and Fear of Cancer Recurrence Among Chinese Breast Cancer Survivors: The Mediation Role of Illness Uncertainty

**DOI:** 10.3389/fpsyg.2022.864129

**Published:** 2022-03-16

**Authors:** Zhichao Yu, Di Sun, Jia Sun

**Affiliations:** ^1^Intensive Care Unit, Shengjing Hospital of China Medical University, Shenyang, China; ^2^School of Nursing, Liaoning University of Traditional Chinese Medicine, Shenyang, China; ^3^Department of Obstetrics, Shengjing Hospital of China Medical University, Shenyang, China

**Keywords:** breast cancer, fear of cancer recurrence, illness uncertainty, social support, structural equation model

## Abstract

**Objective:**

To examine the relations between social support, illness uncertainty (IU), and fear of cancer recurrence (FCR).

**Methods:**

Using data from a convenience sample of 231 breast cancer survivors in China to perform structural equation modeling with bootstrapping estimation. Participants were recruited from a general hospital in Shenyang, China. Participants completed the Perceived Social Support Scale, Mishel Uncertainty in Illness Scale, and Fear of Cancer Recurrence Inventory- Shorter Form.

**Results:**

The majority of breast cancer survivors have FCR (67.5%). FCR was significantly negatively associated with social support, and was significantly positively associated with IU (both *P* < 0.01). Moreover, IU was found to mediate the relationship between social support and FCR (standardized indirect effect = –0.18; bias-corrected 95% confidence interval: –0.255, –0.123).

**Conclusion:**

The findings support the final model. Good social support can directly mitigate FCR, while illness uncertainty can play a mediation role between social support and FCR. Further studies should be conducted to explore effective interventions for social support and IU to ultimately mitigate FCR in cancer survivors.

## Introduction

Cancer is a major public health problem worldwide. Among all types of cancer, breast cancer not only leads the number of women with cancer but also has surpassed lung cancer as the most common cancer. In 2020, the number of new cases of breast cancer in China exceeded 410,000, accounting for approximately 9.1% of all new cancer diagnoses in China and 19.9% of new cancer diagnoses in Chinese women. In other words, breast cancer is diagnosed in 1 out of every 5 newly diagnosed cancer cases in women (Sung et al., 2021). Fortunately, with increased public health awareness, early diagnosis through the mammogram, and improved treatment. The mortality rate for women with breast cancer today is down 42% compared to 1989, and the 5-year relative survival rate for women diagnosed with breast cancer is as high as 90%, resulting in a huge number of breast cancer survivors (Siegel et al., 2022). The psychological problems of breast cancer survivors cannot be ignored, and among them, fear of cancer recurrence (FCR) is the most commonly reported concern and the most frequently recognized unmet need.

Fear of cancer recurrence is defined as the “fear, worry, or concern about cancer returning or progressing (Lebel et al., 2016).” In a study of 240 breast cancer survivors in China, a total of 159 breast cancer survivors (76.81%) were found to have experienced high levels of FCR, characterized by lower functional and overall health than survivors with a low FCR (Peng et al., 2019). According to Schapira et al.’s (2022) study of breast cancer survivors’ FCR trajectories confirmed that although some breast cancer survivors’ FCR improves over time, approximately one-third of breast cancer survivors’ FCR remains severe, even within 5 years of diagnosis. Cancer survivors experiencing FCR primarily present with hypervigilance to changes in physical symptoms and may lead to increased healthcare utilization, resulting in increased personal financial burden and waste of public healthcare resources (Vachon et al., 2021). In addition, FCR has been demonstrated in previous studies to be strongly associated with the development of negative emotions such as anxiety and depression, which without timely intervention may affect the psychological functioning of cancer survivors, resulting in reduced quality of life and even an increased risk of suicide (Zhang et al., 2021).

The mechanisms of FCR triggering and formation are not yet conclusive, and terror management theory (TMT) seems to provide an informed framework for the study of FCR (Simonelli et al., 2017). The core idea of TMT is that human beings have higher cognitive functions and are aware of the finiteness of their lives, thus the survival instinct and mortality consciousness create a unique existence dilemma for human beings, anxiety and fear of existence and death arise when they suffer from death-related stimulation. As a serious life-threatening disease, cancer has the potential to recur even after aggressive treatment. Therefore, based on TMT, we speculated that cancer survivors’ heightened vigilance against cancer recurrence may awaken their sense of mortality eventually leading to FCR. High vigilance for cancer recurrence is mainly reflected in cancer survivors’ over-examination, over-stress and over-attention to changes in physical symptoms, and certain physical symptoms such as pain and chest tightness are considered to be signs of cancer recurrence (Savard and Ivers, 2013). The reason why cancer survivors are overly concerned about physical symptoms may be that, unlike other diseases, the growth and development of cancer cells in the body is usually insidious and not easily detected and controlled, resulting in a strong sense of illness uncertainty (IU) among cancer survivors. IU is a common psychological reaction to the experience of cancer. It is defined as “the inability to determine the meaning of illness-related events” and accurately anticipate or predict health outcomes (Mishel, 1988; Zhang, 2017). However, few studies have been conducted on the relationship between IU and FCR in cancer survivors, which may lead to a lack of direction in FCR intervention programs. Therefore, based on the current state of research, we believe it is necessary to investigate the relationship between IU and FCR to clarify the interaction between each other.

In addition, psychological problems of cancer survivors are inextricably linked to social factors, social support plays an important role in quality of life and health outcomes after breast cancer diagnosis and treatment (Thompson et al., 2017). Previous studies have found a positive effect of social support on ameliorating FCR, and in Niu et al.’s (2019) study of 342 breast cancer patients, social support was found to be an independent predictor of FCR; Hong et al.’s (2020) study also highlighted that successful psychological adjustment of cancer patients receiving chemotherapy may be facilitated by improved social support. The mechanism of action by which social support affects FCR is unclear. Nevertheless, according to Mishel’s (1988, 1990) uncertainty in illness theory, among the antecedents of IU formation, social support, as an important component of structure providers, influences the patient’s understanding of disease-related stimuli and plays a crucial role in the eventual generation of IU. Therefore, we hypothesized that social support may be mediating the impact on FCR through improvements in IU for cancer survivors.

**FIGURE 1:**
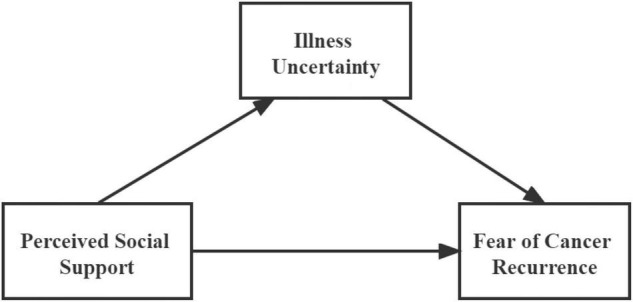
The hypothesized model.

In summary, based on the literature reviewed previously, two hypotheses were proposed: H1, social support is negatively correlated with FCR; H2, IU mediates the effect of social support on FCR. The hypothesized model is illustrated in Figure 1.

## Materials and Methods

### Study Design and Participants

This was a descriptive cross-sectional study, which follows the STROBE guidelines. A convenience sample was recruited from a general hospital in Shenyang, China, between February and September 2021. Inclusion criteria were as follows: (1) survivors with a clinicopathological histological or cytological diagnosis breast cancer; (2) age ≥ 18 years; (3) good reading and communication skills in Chinese; (4) and volunteer for this study. We excluded patients who were diagnosed with other complications and those who were unable to complete the questionnaire due to psychological or cognitive impairment. Depending on the requirements of the structural equation model (SEM), a sample size of between 200 and 400 being appropriate (Hair et al., 1998). We distributed the survey questionnaire to 245 potential participants, ten survivors declined to participate due to health issues or lack of interest, and four survivors were absent when the survey was conducted. Therefore, the final sample comprised 231 participants with an 94.3% overall response rate. The sample size met the requirements for SEM analysis.

### Procedure

The present study uses a Chinese version of the questionnaire to collect information about participants. The questionnaire consisted of four main self-report sections: Sociodemographic Characteristics, Perceived Social Support Scale (PSSS), Mishel Uncertainty in Illness Scale (MUIS), and a shorter form of the Fear of Cancer Recurrence Inventory (FCRI-SF). The assessors used a uniform instruction guide to brief the participants on the purpose and significance of the study and assisted the participants who had difficulty reading the questionnaire. The assessors collected the completed questionnaire on the spot, and asked the participants to fill in any missing options. All participants were given a small gift as compensation for completing the questionnaire.

### Ethics Approval

Ethical approval for this study was obtained from the Ethics Committee of Shengjing Hospital of China Medical University. In accordance with the Declaration of Helsinki, participants were informed of the purpose and procedures of the study before it began. They had the right to leave the study at any time and were not required to answer any questions. All participants were given written informed consent, which indicated that they fully understood the study procedures.

### Measures

#### Sociodemographic Characteristics

A self-designed questionnaire was used to collect sociodemographic characteristics of patients including age, education, marital status, primary caregivers, cancer stages, and with or without breast cancer recurrence.

#### Perceived Social Support Scale

The social support was assessed using the Chinese version PSSS, which was developed by Zimet (Blumenthal et al., 1987) and modified by Jiang (Huang et al., 1996). It consists of 12 items, including three dimensions, namely, family support, friend support, and other support, each containing four items. Each item was scored on seven-point Likert scales (ranging from 1 to 7). The higher the score, the higher the level of social support as perceived by the individual. A total score between 12 and 36 is considered low support, 37–60 is moderate support, and 61–84 is high support. The Cronbach’s α for the total scale was 0.88 (Huang et al., 1996). Cronbach’s α in the current sample was 0.92.

#### Mishel Uncertainty in Illness Scale

The IU was assessed using the Chinese version MUIS, which was developed by Mishel (1997) and modified by Xu and Huang (1996). It consists of 25 items and includes two dimensions: ambiguity (15 items) and complexity (10 items). These items were scored on five-point Likert scales, ranging from strongly disagree to strongly agree. The scale scores range from 25 to 125 and can be divided into three levels, low level 25–58, medium level 59–91, and high level 92–125. Higher scores indicate higher levels of uncertainty about the patient’s illness. The content validity index (CVI) was 0.92 and Cronbach’s α was 0.87 (Xu and Huang, 1996). In the current sample, Cronbach’s α was 0.86.

#### Fear of Cancer Recurrence Inventory-Shorter Form

The FCR was assessed using the Chinese version FCRI-SF, which was developed by Simard and Savard (2015) and modified by Su et al. (2018). It consists of nine items, which were scored on five-point Likert scales (ranging from 0 to 4). Cancer patients with a score greater than 13 may have FCR. The Cronbach’s α was 0.90. Cronbach’s α in the current sample was 0.79.

### Statistical Analysis

Descriptive statistics were used to describe the sociodemographic characteristics of the participants and the main study variables (PSS, IU, and FCR). In addition, tests for normality and homogeneity of variance were performed. One-way ANOVAs and *t*-tests were used to determine the relationship between participant characteristics and the three variables, and Pearson correlations were used to test for unadjusted associations between variables. These data above were statistically analyzed using IBM SPSS Statistics 26.0 (IBM Corporation, United States). The hypothetical model was tested using SEM with IBM SPSS AMOS version 26.0 (IBM Corporation, United States). The maximum-likelihood estimation of the entire system in a hypothesized model, and enables the assessment of variables with the data (Jöreskog and Sörbom, 1982). Finally, bootstrap tests were used to measure the direct, indirect and total effects of the model (Hayes, 2009). Statistical significance was set at 0.05.

## Results

### Sociodemographic and Psychosocial Characteristics

A total of 231 females with breast cancer participated in this study. The descriptive statistics for all study variables are shown in Table 1. The mean age of breast cancer patients was 52 years (standard deviation [SD] = 11), with a range of 31–82 years. The majority were married (80.5%), junior high school or less (36.4%), spouse of the patients (42.0%), breast cancer stage II (53.7%), no recurrence (83.1%), moderate social support (55.4%) and IU (78.8%), and FCR scores more than 13 (67.5%). The mean scores for PSSS, MUIS and FCRI-SF were 58.83 (SD = 10.94), 66.26 (SD = 10.95), and 14.19 (SD = 5.31).

**TABLE 1:** Sociodemographic and psychosocial characteristics (*n* = 231).

	*n*	%	M	SD	Range
Age			52	11	31–82
**Marital status**					
Married	186	80.5			
Other	45	19.5			
**Education**					
Junior high school or less	84	36.4			
High school	74	32.0			
Bachelor and above	73	31.6			
**Primary caregivers**					
Spouse	97	42.0			
Parents	11	4.8			
Children	68	29.4			
Relatives and friends	36	15.6			
Attendant	19	8.2			
**Cancer stages**					
I	34	14.7			
II	124	53.7			
III	55	23.8			
IV	18	7.8			
**Recurrence**					
Yes	39	16.9			
No	192	83.1			
**PSSS Total**			58.83	10.94	35–84
Family support			20.61	4.27	10–28
Friend support			18.76	4.12	11–28
Other support			19.46	3.96	11–28
Low	2	0.9			
Moderate	128	55.4			
High	101	43.7			
**MUIS Total**			66.26	10.95	25–100
Ambiguity			40.77	7.53	15–73
Complexity			25.49	5.24	10–37
Low	48	20.8			
Moderate	182	78.8			
High	1	0.4			
**FCRI-SF Total**			14.19	5.31	3–32
≥13	156	67.5			
<13	75	32.5			

*M, mean; SD, standard deviation; PSSS, Perceived Social Support Scale; MUIS, Mishel Uncertainty in Illness Scale; FCRI-SF, Fear of Cancer Recurrence Inventory-Shorter Form.*

### Social Support, Illness Uncertainty, and Fear of Cancer Recurrence, According to Sample Characteristics

As shown in Table 2, survivors aged ≤35 years reported significantly higher FCRI-SF scores than those aged 36–59 years and ≥60 years (*F* = 7.308, *P* < 0.01). Regarding primary caregivers, survivors whose care was provided by an attendant reported lower PSSS scores and higher MUIS and FCRI-SF scores than compared with those whose care was provided by spouse, parents, children, relatives, and friends (*F* = 5.127, *P* < 0.01; *F* = 2.995, *P* < 0.05; *F* = 2.654, *P* < 0.05). Survivors with recurrent breast cancer reported higher FCRI-SF scores than those without recurrence (*t* = 3.191, *P* < 0.01).

**TABLE 2:** Social support, IU, and FCR, according to sociodemographic characteristics (*n* = 231).

Outcomes	PSSS	MUIS	FCRI-SF
**Age**			
≤35	59.45 ± 11.48	66.14 ± 14.78	17.86 ± 7.95^a^
36–59	58.11 ± 11.16	66.46 ± 9.69	13.45 ± 4.73
≥60	60.27 ± 10.21	65.86 ± 12.27	14.63 ± 4.95
*F*	0.897	0.068	7.308
*P*	0.409	0.935	0.001**
**Education**			
Junior high school or less	61.07 ± 8.69	64.93 ± 11.31	13.63 ± 5.33
High school	57.62 ± 12.44	65.91 ± 10.23	13.68 ± 4.49
Bachelor and above	57.47 ± 11.35	68.16 ± 11.10	15.36 ± 5.90
*F*	2.827	1.777	2.611
*P*	0.061	0.170	0.076
**Marital status**			
Married	58.95 ± 11.33	65.84 ± 11.19	14.08 ± 5.28
Other	58.31 ± 9.22	68.02 ± 9.77	14.64 ± 5.48
*t*	0.352	–1.202	–0.639
*P*	0.725	0.231	0.524
**Primary caregivers**			
Spouse	60.33 ± 11.57	65.66 ± 9.64	13.19 ± 5.55
Parents	61.27 ± 9.79	62.09 ± 14.41	14.27 ± 4.43
Children	60.22 ± 9.23	64.84 ± 12.73	14.35 ± 4.74
Relatives and friends	56.31 ± 11.16	68.28 ± 10.17	15.00 ± 5.09
Attendant	49.53 ± 8.64^b^	73.05 ± 6.16^a^	17.16 ± 5.86^a^
*F*	5.127	2.995	2.654
*P*	0.001**	0.020*	0.034*
**Cancer stages**			
I	61.12 ± 9.57	67.62 ± 12.96	13.12 ± 5.04
II	57.17 ± 11.72	67.51 ± 9.49	14.47 ± 5.27
III	60.65 ± 9.21	63.31 ± 12.05	13.60 ± 5.15
IV	60.33 ± 11.56	64.17 ± 11.62	16.11 ± 6.22
*F*	2.102	2.303	1.601
*P*	0.101	0.078	0.190
**Recurrence**			
Yes	57.41 ± 11.27	69.23 ± 10.08	16.62 ± 6.21
No	59.11 ± 10.88	65.66 ± 11.04	13.70 ± 4.98
*t*	-0.887	1.867	3.191
*P*	0.376	0.063	0.002**

*P < 0.05, **P < 0.01. ^a^Post-hoc tests showed the score of this group was higher than the scores of the other groups. ^b^Post-hoc tests showed the score of this group was lower than the scores of the other groups. IU, Illness Uncertainty; FCR, Fear of Cancer Recurrence; PSSS, Perceived Social Support Scale; MUIS, Mishel Uncertainty in Illness Scale; FCRI-SF, Fear of Cancer Recurrence Inventory-Shorter Form.

### The Correlations Between Social Support, Illness Uncertainty, and Fear of Cancer Recurrence

Pearson’s correlation analysis (Table 3) showed that FCR (i.e., FCRI-SF total scores) were significantly and negatively correlated with social support (i.e., PSSS total and dimensions scores) (*r* = –0.31 to –0.38, *P* < 0.01). FCR were significantly and positively correlated with IU (i.e., MUIS total and dimensions scores) (*r* = 0.40 to 0.54, *P* < 0.01). Furthermore, social support was significantly and negatively associated with IU (*r* = –0.21 to –0.51, *P* < 0.01).

**TABLE 3:** Correlations (*r*) between social support, IU, and FCR (*n* = 231).

	1	2	3	4	5	6	7	8
1. PSSS Total	1							
2. Family Support	0.90**	1						
3. Friend Support	0.84**	0.59**	1					
4. Other Support	0.92**	0.79**	0.66**	1				
5. MUIS Total	−0.40**	−0.39**	−0.29**	−0.37**	1			
6. Ambiguity	−0.24**	−0.21**	−0.21**	−0.21**	0.90**	1		
7. Complexity	−0.48**	−0.51**	−0.29**	−0.46**	0.79**	0.45**	1	
8. FCRI-SF Total	−0.38**	−0.36**	−0.31**	−0.34**	0.54**	0.50**	0.40**	1

**P < 0.01. IU, Illness Uncertainty; FCR, Fear of Cancer Recurrence; PSSS, Perceived Social Support Scale; MUIS, Mishel Uncertainty in Illness Scale; FCRI-SF, Fear of Cancer Recurrence Inventory- Shorter Form.

### Common Method Variance

As with all self-reported data, there is the potential for common method variance (CMV) leading from multiple sources (Podsakoff et al., 2003). As a result, we performed a Harmon one-factor test was conducted on the 46 items in our hypothetical model (Podsakoff and Organ, 1986). According to Jukka (2009), the first factor tends to explain over half of the variance, indicating the presence of CMV. The results showed that 12 factors are present and the most covariance explained by one factor is 24.00 percent, indicating that CMV is not a likely contaminant of our results.

### Test of the Model

**FIGURE 2:**
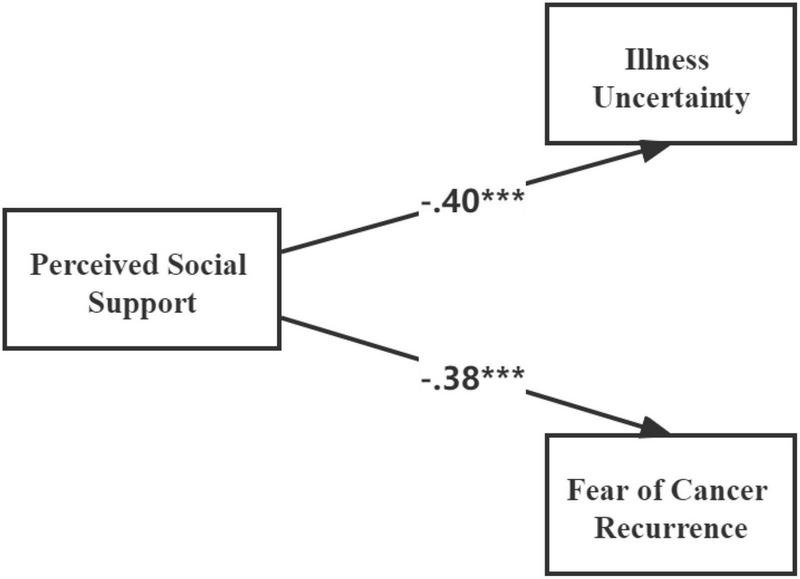
Direct effects of social support on fear of cancer recurrence and illness uncertainty. ***P < 0.001 (two-tailed); *n* = 231.

We use a causal step strategy to investigate the first mediation condition with respect to hypothesis 1 (Baron and Kenny, 1986). As shown in Table 3, the correlation coefficients indicated that social support was significantly and negatively associated with FCR (*r* = –0.38, *P* < 0.01). Additionally, the results of the direct effect of social support on FCR (standardized direct effect = –0.38, *P* < 0.001, see Figure 2) was statistically significant. Hypotheses 1 was thus supported.

**FIGURE 3:**
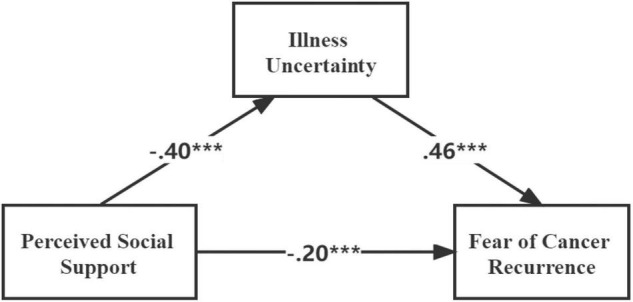
Structural equation modeling of the hypothesized model. ***P < 0.001 (two-tailed); *n* = 231.

To test hypothesis 2, we measured the second condition of mediation. The correlation coefficients indicated that social support was significantly and negatively associated with IU (*r* = –0.40, *P* < 0.01), IU was significantly and positively associated with FCR (*r* = 0.54, *P* < 0.01). In addition, the results of the direct effects of social support on IU (standardized direct effect = –0.40, *P* < 0.001), and the direct effect of IU on FCR (standardized direct effect = 0.46, *P* < 0.001, see Figure 3), were all statistically significant. To investigate the indirect effects of the dependent variable through mediation, we conducted bias-corrected percentile bootstrapping and percentile bootstrapping with 5,000 bootstrap samples at 95% confidence interval (Table 4; MacKinnon, 2008). We calculated the confidence interval of the lower and upper bounds to test whether the indirect effects were significant (Wang et al., 2014). The result of the bootstrap test confirmed the existence of a significant mediating effect for IU between social support and FCR (standardized indirect effect = –0.18, *P* < 0.01). Therefore, hypothesis 2 was supported.

**TABLE 4:** Standardized direct, indirect, and total effects of the hypothesized model.

	Point estimate	Product of coefficients		Bootstrapping				
				Bias-corrected 95% CI		Percentile 95% CI		Two-tailed significance
		SE	Z	Lower	Upper	Lower	Upper	
**Direct effects**								
SS → FCR	−0.200	0.057	−3.509	−0.309	−0.086	−0.311	−0.088	0.001**
**Indirect effects**								
SS → FCR	−0.181	0.033	−5.485	−0.255	−0.123	−0.251	−0.121	0.000***
**Total effects**								
SS → FCR	−0.381	0.048	−7.938	−0.473	−0.284	−0.475	−0.285	0.000***

Estimating of 5,000 bootstrap sample, **P < 0.01, ***P < 0.001. SS, Social Support; FCR, Fear of Cancer Recurrence.

## Discussion

The purpose of this study was to investigate the interactions between social support, IU, and FCR. The results of this study showed that there was a negative association between social support and IU and FCR, while a positive association was presented between IU and FCR. The findings further emphasize the importance of social support in promoting the development of psychological wellbeing in breast cancer survivors. Similar results have been found in previous studies (Taha et al., 2012; Hamama-Raz et al., 2021). Furthermore, to our knowledge, the present study is the first theoretically based and empirical study to examine whether IU mediates the negative relationship between social support and FCR.

Fear of cancer recurrence is recognized as the most common psychological problem among cancer survivors, and the FCR score of breast cancer survivors in this study was found to be 14.19 ± 5.31. According to the diagnostic threshold of FCRI-SF, 67.5% of breast cancer survivors in this study had FCR, and the occurrence of FCR is not optimistic. Although there is no consensus on the clinical cutoff value for FCR, current findings generally suggest that the incidence of FCR is gender-related and that women are more likely to develop FCR (Tauber et al., 2019; Borreani et al., 2020; Muldbücker et al., 2021). Breast cancer, as the most prevalent type of cancer in women, suggests that healthcare providers should increase screening for FCR in this group to achieve early identification, diagnosis, and intervention to avoid adverse outcomes due to FCR. The results of this study found that approximately close to 80% of breast cancer survivors had moderate to high levels of IU, suggesting that IU may be prevalent. Consistent results were also reported in a study of breast cancer survivors by Hagen et al. (2015). Breast cancer survivors may need to undergo one or more treatments including surgery, chemotherapy, radiation, endocrine therapy, and biologic therapy. Not only that, post-treatment survivors face complex rehabilitation and self-care. As a result, survivors who lack the medical background often appear overwhelmed and eventually develop IU. Finally, this study found that almost all breast cancer survivors perceived moderate to high levels of social support, which supports the previous study’s conclusion (Sørensen et al., 2020). Social support can be classified according to its source as endogenous family social support and exogenous family social support. The emphasis on family is an important feature of Chinese culture, while family support is generally considered to be dominant within the social support system. The predominance of spouses and children as caregivers for the breast cancer survivors included in this study also provides some evidence of the unique position of family in the Chinese heart, and it may also be the reason for the overall high level of social support in the results.

When social support, IU and FCR were examined according to sociodemographic characteristics, the differences in partial PSSS, MUIS, and FCRI-SF were significant. The present study, survivors aged less than 35 years reported higher levels of FCR. Similarly, Lane et al. (2019) found that adolescent patients generally had higher levels of FCR than middle-aged and older patients. Younger survivors are usually at a critical stage of growth in life, with more work and family responsibilities, and may face more financial and social challenges and fear negative career and family consequences due to cancer recurrence, while older survivors have more experience and exposure, are more resilient to stress and have more positive emotional responses. As a result, younger survivors are likely to have higher FCR levels. In terms of primary caregivers, survivors who were cared for by attendant perceived lower levels of social support and higher levels of IU and FCR than survivors whose spouses, parents, children, relatives and friends provided care. This may be due to the influence of the employment relationship. Attendant only provide care for survivors and are less intimate with survivors, less emotionally invested in survivors and more concerned with their own interests than the survivors’ families. However, kinship relationships are unchangeable and care from spouses, parents and children can give more support to survivors. In addition, breast cancer survivors who have experienced cancer recurrence have higher levels of FCR, which may be due to the length of time it takes to treat the cancer and the severe side effects of the treatment. The painful memory of the experience adds to the fears of cancer survivors who have experienced recurrence.

In line with our proposed hypothesis 1, this study found a negative direct effect between social support and FCR, suggesting that breast cancer survivors with higher levels of social support were less vulnerable to experiencing FCR. This result is generally consistent with the findings of a recently published evidence-based study (Zhang et al., 2021). According to the social support buffering model, social support functions as a buffer between the subjective experience of stress and illness (Cohen and Wills, 1985). Breast cancer survivors need to be constantly alert to the risk of cancer recurrence, and the prolonged state of high alertness inevitably generates severe psychological stress resulting in fear of recurrence. The timely intervention of social support can provide survivors with more problem-solving strategies and reduce the importance of the problem, thus reducing the adverse effects of stress such as anxiety, depression, and FCR. In addition to its contribution to the psychological wellbeing of cancer survivors, social support may also have a positive impact on patient survival. A Meta-analysis of 106 randomized controlled trials by Smith et al. (2021) found that psychosocial support fostered motivation to exercise and encouraged patients to complete treatment, and increased overall patient survival by 29%. Therefore, given the positive impact of social support on the physical and mental health of cancer survivors, it is necessary to implement some effective interventions to improve the level of social support for cancer survivors. However, it is worth noting that because each cancer survivor may have different personality traits, cultural environment, and nature of stressors, their needs for types of support may be different. For example, a qualitative study by Korotkin et al. (2019) found that more anxious cancer survivors were more likely to want companionship support, while younger cancer survivors were more likely to want home care support. This suggests that healthcare professionals should ideally individualize their social support interventions to meet the psychological needs of different cancer survivors in order to maximize the benefits of the intervention.

The results of the present study confirm our proposed second hypothesis that IU plays a partially mediating role in the negative effect of social support on FCR. This indicates that when breast cancer survivors are faced with a stressful disease event and inadequate social support, they first develop IU, and the stimulus of IU is perceived by cancer survivors as a threat of death that ultimately causes them to experience FCR. The results highlight the importance of IU in the development of FCR and validate the antecedent framework of Mishel’s (1988) uncertainty in illness theory. Previous studies have also reported a negative association between social support and IU similar to the results of this study (Lee and Park, 2020). Cancer survivors’ perceptions and interpretations of stressful disease events are often considered to be key factors in the occurrence of IU (Zhang, 2017). In contrast, social support can act as an intermediate link between stressful events and subjective appraisals (Cohen and Wills, 1985). Social support can enable cancer survivors to more accurately predict and understand their experiences, will underestimate the damaging nature of stressful scenarios, and reduce IU by improving subjective perceptions and self-coping skills and reducing evaluations of the severity of stressful illness events. It seems that the direction of social support for managing IU and FCR in cancer survivors could be in terms of increasing physician-patient communication and facilitating the exchange of disease-related information. Effective physician-patient communication has been shown to have a positive effect on reducing IU and FCR (Dawson et al., 2016). Hence, as cancer gradually becomes a chronic disease, healthcare professionals, by providing cancer survivors with disease-related information and teaching self-health management skills will help survivors to re-evaluate their disease status to a great extent, enabling them to gain motivation to accept and adhere to rehabilitation treatment, which promotes psychological adaptation to avoid IU and FCR.

### Limitations

The present study has a number of limitations. First, this study was cross-sectional and although we used SEM to conduct a simultaneous testing of our proposed model in breast cancer survivors, the results still need to be treated with caution. It is suggested that future studies could address this issue by obtaining longitudinal data to strengthen the causal relationship between social support, IU, and FCR. Second, our data came from self-reports, which raises the possibility of CMV. However, as the variables examined in this study, namely, social support, IU, and FCR, reveal the psychological state of the individual, there is a sound theoretical basis for collecting these data from the survivors themselves. In addition, statistical analyses were conducted to reduce or prevent the potential impact of CMV. Finally, this study was a single-center study that used convenience sampling to recruit participants and therefore limited generalization due to potential selection bias. It is hoped that multi-center and random sample studies will emerge in the future.

## Conclusion

This preliminary study supports the final model. Good social support can directly mitigate FCR, while illness uncertainty can play a mediation role between social support and FCR. Further studies should be conducted to explore effective interventions for social support and IU to ultimately mitigate FCR in cancer survivors.

## Data Availability Statement

The raw data supporting the conclusions of this article will be made available by the authors, without undue reservation.

## Ethics Statement

The studies involving human participants were reviewed and approved by Ethics Committee of Shengjing Hospital of China Medical University. The patients/participants provided their written informed consent to participate in this study.

## Author Contributions

JS and ZCY conceptualized the study, and contributed to final analysis and interpretation of the data. ZCY and JS undertook data collection and preliminary analysis assisted by DS. All authors developed the methodology and analytical plan, read, and approved the final manuscript.

## Conflict of Interest

The authors declare that the research was conducted in the absence of any commercial or financial relationships that could be construed as a potential conflict of interest.

## Publisher’s Note

All claims expressed in this article are solely those of the authors and do not necessarily represent those of their affiliated organizations, or those of the publisher, the editors and the reviewers. Any product that may be evaluated in this article, or claim that may be made by its manufacturer, is not guaranteed or endorsed by the publisher.
